# Cranial Irradiation Alters the Brain’s Microenvironment and Permits CCR2^+^ Macrophage Infiltration

**DOI:** 10.1371/journal.pone.0093650

**Published:** 2014-04-02

**Authors:** Josh M. Morganti, Timothy D. Jopson, Sharon Liu, Nalin Gupta, Susanna Rosi

**Affiliations:** 1 Brain and Spinal Injury Center, University of California San Francisco, San Francisco, California, United States of America; 2 Departments of Physical Therapy and Rehabilitation Science, University of California San Francisco, San Francisco, California, United States of America; 3 Neurological Surgery, University of California San Francisco, San Francisco, California, United States of America; National Taiwan University, Taiwan

## Abstract

Therapeutic irradiation is commonly used to treat primary or metastatic central nervous system tumors. It is believed that activation of neuroinflammatory signaling pathways contributes to the development of common adverse effects, which may ultimately contribute to cognitive dysfunction. Recent studies identified the chemokine (C-C motif) receptor (CCR2), constitutively expressed by cells of the monocyte-macrophage lineage, as a mediator of cognitive impairments induced by irradiation. In the present study we utilized a unique reporter mouse (*CCR2^RFP/+^CX3CR1^GFP/+^*) to accurately delineate the resident (CX3CR1^+^) versus peripheral (CCR2^+^) innate immune response in the brain following cranial irradiation. Our results demonstrate that a single dose of 10Gy cranial γ-irradiation induced a significant decrease in the percentage of resident microglia, while inducing an increase in the infiltration of peripherally derived CCR2^+^ macrophages. Although reduced in percentage, there was a significant increase in F4/80^+^ activated macrophages in irradiated animals compared to sham. Moreover, we found that there were altered levels of pro-inflammatory cytokines, chemokines, adhesion molecules, and growth factors in the hippocampi of wild type irradiated mice as compared to sham. All of these molecules are implicated in the recruitment, adhesion, and migration of peripheral monocytes to injured tissue. Importantly, there were no measureable changes in the expression of multiple markers associated with blood-brain barrier integrity; implicating the infiltration of peripheral CCR2^+^ macrophages may be due to inflammatory induced chemotactic signaling. Cumulatively, these data provide evidence that therapeutic levels of cranial radiation are sufficient to alter the brain’s homeostatic balance and permit the influx of peripherally-derived CCR2^+^ macrophages as well as the regional susceptibility of the hippocampal formation to ionizing radiation.

## Introduction

Ionizing radiation is commonly used to treat both primary and metastatic brain tumors and can cause a number of late effects including progressive cognitive dysfunction [1]. Specifically, irradiation of the temporal lobe can profoundly affect the cellular structures mediating learning and memory [2]–[4]. Ionizing radiation has been consistently shown to affect multiple neuroinflammatory signaling cascades [5]–[7] ultimately causing disruptions in hippocampal function [3]–[5], [8], [9]. Importantly, broad-spectrum anti-inflammatory treatment has been shown to abrogate certain aspects of radiation-induced hippocampal functional deficits [4], [9]. We have recently shown that radiation-induced disruption in neuronal networks associated with learning and memory as well as inflammatory response are blunted in CCR2-deficient mice following cranial radiation [10]. Previously, CCR2 has been shown to be expressed on neurons and glia [11], [12], although more recent evidence suggests that CCR2 is expressed predominantly on blood-born monocytes and macrophages rather than resident cells in the central nervous system (CNS) [13]–[17].

Monocytes are important mediators of innate immune function given their ability to differentiate into tissue macrophages [18]–[20]. Recent work has demonstrated that monocytes can be divided into two distinct subpopulations based upon expression of specific cell surface antigens. Notably, these are termed ‘inflammatory’ (Ly-6C^hi^CCR2^+^CX3CR1^−^) and ‘circulating’ (Ly-6C^l^°CCR2^−^CX3CR1^+^) monocytes. Monocytes expressing the chemokine receptor CCR2 (i.e. inflammatory monocytes) are able to migrate from bone marrow, infiltrate injured tissues where they become macrophages, and produce high levels of pro-inflammatory cytokines [18], [21]–[23]. It has been shown that the role of these subpopulations may differ between various models of disease [24], [25]. Interestingly, blocking CCR2 signaling has been shown to be both neuroprotective or neurotoxic in various animal models of neurodegenerative disease [16], [21], [26]–[32].

Multiple studies using different animals models have shown that CNS resident microglia do not express CCR2 *in vivo* [14]–[17]. Moreover, in a mouse model of stroke using CCR2-deficient mice, there is no difference in resident microglia activation, suggesting that CCR2 does not regulate resident microglial response [33]. Additionally, *in vitro* studies have shown that isolated mouse microglia lack significant expression of CCR2 mRNA [34], [35]. However, CCR2 depletion has been shown to affect the emigration of bone-marrow derived monocytes into circulation, ultimately reducing the infiltration into CNS tissues in various models of neurological disease [14], [17], [21], [33], [36]. Cumulatively, these studies suggest that the expression of CCR2 is predominantly attributed to bone-marrow derived circulating monocytes.

Cranial irradiation induces pleiotropic neuroinflammatory signaling cascades in multiple rodent models [7], [37], which can be abrogated by broad spectrum anti-inflammatory treatments [4], [9], implicating the innate immune response in the brain. However, the effect of cranial radiation on microglia, the brain’s resident tissue macrophage, remains ambiguous in the context of short-term alterations after irradiation. Moreover, the role of CCR2^+^ macrophage infiltration following cranial irradiation remains unclear.

In the present study we examined the response of resident microglia after cranial irradiation in addition to multiple signaling factors associated with neuroinflammation to determine if irradiation induces a permissive environment for infiltrating CCR2^+^ macrophages. To separate resident microglia from macrophages derived from circulating monocytes *in vivo*, we utilized a unique reporter mouse (*CCR2^RFP/+^CX3CR1^GFP/+^*) to accurately delineate cells based on their expression of either CX3CR1^+^ or CCR2^+^ following cranial irradiation. The genotype of these mice allows for characterization by flow cytometry of macrophages derived from a peripheral source (CCR2^+^) from those derived from cells residing in the CNS (CX3CR1^+^). Using our established animal model [5], [10] in conjunction with flow cytometry and multiple biochemical analyses, our results demonstrate that cranial irradiation (10 Gy) alters the pool of resident microglia as well as multiple pro-inflammatory mediators to create a permissive environment for the infiltration of CCR2^+^ macrophages into the brain parenchyma.

## Methods

### Animals

All experiments were conducted in accordance with the National Institutes of Health Guide for the Care and Use of Laboratory Animals and were approved by the Institutional Animal Care and Use Committee of the University of California (San Francisco, CA). Three-month-old male *C57BL/6J* (WT) and *CCR2^RFP/+^CX3CR1^GFP/+^* mice were used for the proceeding experiments. WT mice were purchased from The Jackson Laboratory. *CCR2^RFP/+^CX3CR1^GFP/+^*mice were generated as previously described [16] and genotype was confirmed using a commercially available service (Transnetyx; Cordova, TN). Mice were group housed in environmentally controlled conditions (12∶12 h light:dark cycle at 21±1°C) and provided food and water *ad libitum*.

### Radiation Procedure

Animals were randomly assigned to receive either cranial radiation or sham procedures, as we have previously described [10]. The eyes and body were shielded by a lead collimator, which limited the beam to a width of 1.0 cm. An additional lead shield was positioned to block exposure to the trachea. Irradiated animals received a beam exposure to both hemispheres at 5 Gy each, for a total of 10 Gy dose to the head. Sham animals were exposed to identical procedures, however radiation was omitted.

### Lectin Procedure

Seven days following sham or irradiation procedures a small cohort of mice were injected with 100 uL of 5 mg/mL rhodamine-conjucated lectin (Vector #RL-1082) via the tail vein. Twenty minutes following injection, animals were lethally overdosed with an i.p. injection of ketamine/xylazine to allow transcardial perfusion. Mice were transcardially perfused with buffered saline followed by 4% paraformaldehyde in buffered saline.

### Biochemical Analyses Tissue Preparation

Seven days following radiation exposure WT mice were killed via cervical dislocation. Brain tissues encompassing the hippocampus (HPC) were dissected and rapidly frozen in dry ice chilled (−70°C) isopentane before final storage at −80°C. Hippocampi were allocated for either Western blot and ELISA or quantitative reverse transcription PCR (qRT-PCR) analyses. For Western blot and ELISA analyses tissues were prepared as previously described [38]. For qRT-PCR analyses, tissues were homogenized in Qiazol reagent (Qiagen #79306) and RNA was isolated using RNEasy mini-columns (Qiagen #74106) following manufacturer’s suggested protocol. One microgram of total RNA was reverse-transcribed using the High-Capacity cDNA Reverse Transcription Kit (Applied Biosystems #12574035).

### Western Blot Analyses

Fifty micrograms of total protein per lane was loaded onto a 4–15% SDS-polyacrylamide gel (Bio-Rad #567–1084 ) for electrophoresis. Proteins were transferred onto a nitrocellulose membrane for immunodetection. Membranes were subsequently blocked for 1 h in 5% nonfat dry milk (NFDM; Bio-Rad #170–6404) in phosphate buffered saline with Tween20 (PBS-T; 0.1% Tween 20). Antibodies specific for JAM1 (Millipore #04–593, 1∶500), ZO-1 (Invitrogen #40–2200, 1∶500), PECAM1 (AbCam #28364, 1∶500), Claudin5 (Millipore #ABT45,1∶800) and GAPDH (Sigma #G8795, 1∶10,000) were incubated overnight at 4°C in 1% NFDM in PBS-T. Following washes, appropriate secondary antibodies (Li-Cor) were incubated for 1 h at room temperature in 1% NFDM in PBS-T. Membranes were scanned using Li-Cor Odyssey near-infrared imager and raw intensity for each band was measured using Li-Cor Odyssey image analysis software. Scanned pseudo-colored images were converted to black and white.

### ELISA Analyses

Tumor necrosis factor alpha (TNFα, Raybiotech #ELM-TNF-001) and chemokine C-C motif ligand (CCL2, Raybiotech #ELM-MCP1-001C) concentrations were quantified using standard ELISA technique. HPC lysates were run in duplicate at a concentration of 75 μg per well and incubated overnight at 4°C. Following incubation, the manufacturer’s (RayBiotech) suggested protocol was followed. Optical density values for each ELISA were measured on a plate reader and sample concentrations were calculated based upon the supplied standard curve. Values were converted from pg/mL to pg/μg of total protein loaded.

### qRT-PCR Analyses

Quantification of multiple gene transcripts was conducted as previously described [10]. The following primers were used (5′ to 3′ S/AS); *Hif1α:* GATGACGGCGACATGGTTTAC/CTCACTGGGCCATTTCTGTGT, *COX-2*: GCTGTACAAGCAGTGGCAAA/GCTCGGCTTCCAGTATTGAG, *CD68*: GACCTACATCAGAGCCCG/CGCCATGAATGTCCACTG, *CD11b*: CTGAGACTGGAGGCAACCAT/GATATCTCCTTCGCGCAGAC, *GFAP*: ATTGCTGGAGGGCGAAGAA/CGGATCTGGAGGTTGGAGAA, *VEGFa*: ACCATGAACTTTCTGCTCTCTTG/GAACTTGATCACTTCATGGGACT, *VEGFr2*: TGCCTACCTCACCTGTTTCC/CTCTTTCGCTTACTGTTCTGGAG, *ICAM-1*: AAACCAGACCCTGGAACTGCAC/GCCTGGCATTTCAGAGTCTGCT.

Amplifications were carried out in duplicate and the relative expression of target genes was determined by the 2^−ΔΔCt^ method and normalized against cyclophilin gene expression. In each PCR analysis, template and RT controls were included to account for reagent contamination.

### Flow Cytometry

WT and *CCR2^RFP/+^CX3CR1^GFP/+^*mice were lethally overdosed with ketamine (150 mg/kg)/xylazine (15 mg/kg) mixture and transcardially perfused with ice-cold Hank’s balanced salt solution without calcium and magnesium (HBSS). Following perfusion, mice were decapitated and brain hemispheres were separated to either ice-cold RPMI-1640 medium (without phenol; RPMI) or 4% paraformaldehyde (PFA) in buffered saline. Brain hemispheres in RMPI were used for microglia/macrophage isolation following standard procedures [39], while 4% PFA post-fixed tissues were used for sectioning and imaging (see below). Fc receptor blocking was performed before all staining procedures using an anti-CD16/32 antibody (BD Pharmigen #553142). The following reagents were used for labeling isolated microglia/macrophage: 7AAD (Sigma-Aldrich #A9400), CD11b Alexafluor 700 (BD Pharmigen #557960), F4/80 APC (Invitrogen #MF48005). Mandibular blood draws from naïve *CCR2^RFP/RFP^CX3CR1^GFP/GFP^* mice were used as positive controls for RFP and GFP expression. Additionally, naïve WT isolated microglia/macrophages served as negative control for RFP and GFP expression. Spectral compensation was achieved using polystyrene microparticles (BD Pharmigen #552845) in combination with each of the above listed conjugated antibodies following manufacturer’s suggested protocol. Standard staining procedures were conducted as previously described [39] before analysis on FACSAria III cell sorter (BD Biosciences). All samples were diluted 1∶10 and run in duplicate.

### Brain Tissue Sectioning and Imaging

All brain tissue used for fluorescence imaging was sectioned as previously described [38]. 40 μm free-floating sections were mounted onto Superfrost Plus slides (Fisher #12-550-15) and allowed to dry overnight. Slides were rinsed in buffered saline solution before counterstaining with 4′,6-diamidino-2-phenylindole (DAPI; Invitrogen #D1306) followed by coverslipping in Vectashield fluorescent mounting medium (Vector #H1000). All imaging was achieved using a Zeiss Imager.Z1 Apotome microscope controlled by ZEN software (Zeiss 2012).

### Data Analysis

All data were analyzed using Prism software (v6.0, GraphPad; La Jolla, CA) and are presented as the mean ± standard error of the mean (SEM). Statistical analyses were performed using ANOVA or Student’s t-test. Pairwise comparisons within ANOVA were assessed by Tukey’s HSD *post hoc* multiple comparisons test. Throughout, *p* values of <0.05 were considered significant.

## Results

### Cranial Irradiation Induces a Protracted Decrease in CD11b^+^ Myeloid Cells in the Brain

**Figure 1:**
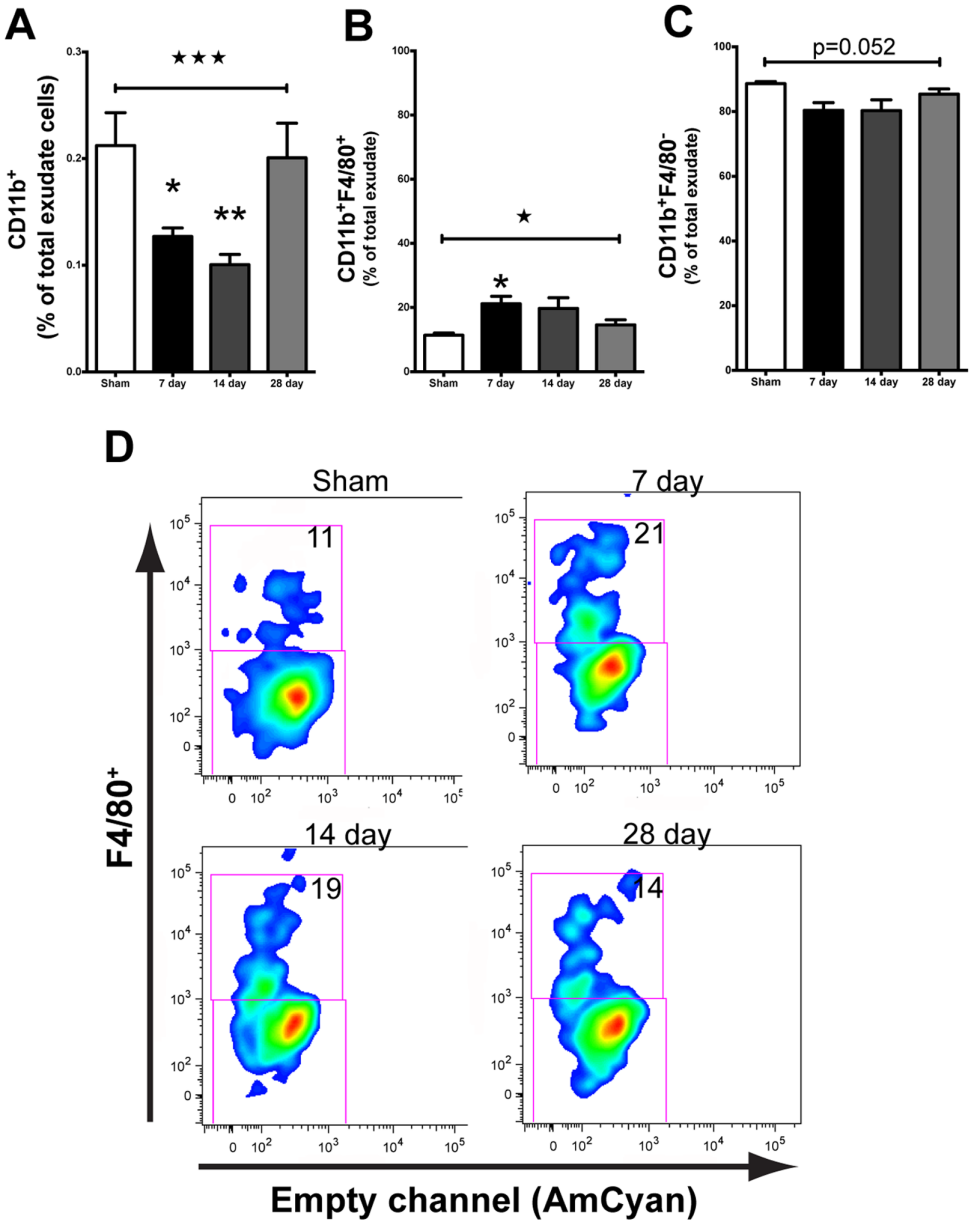
Cranial irradiation alters myeloid cell population over time. **A.** 10^+^ cells (ANOVA; *F_(3,12)_ = 12.07, ^★★★^p = 0.0006*) at 7 (Tukey’s HSD; *p<0.05) and 14 (Tukey’s HSD; **p<0.01) days post irradiation. The percentage of CD11b^+^ cells returned to sham levels by day 28 (Tukey’s HSD; *p>0.05*). **B.** Overall, cranial irradiation induced a significant increase in the percentage of CD11b^+^F4/80^+^ macrophages at 7 days (ANOVA; *F(3,12) = 4.048, ^★^p = 0.0335*; Tukey’s HSD; *p<0.05). **C.** Concomitant with the radiation induced increase of percentage of F4/80^+^ macrophages, we observed a trend for decreased F4/80^−^ stained cells. **D.** Representative plots showing the 10 Gy radiation induced shift in the percentage of CD11b^+^ cells that stained for F4/80 as a function of time. Average proportion of F4/80^+^ is given.

We characterized the response of myeloid cells at 7, 14, and 28 days following ionizing radiation. All mice tolerated cranial radiation (10 Gy) dosage and gained weight normally over the duration of the studies (data not shown) as we have previously reported [5], [10]. A single 10 Gy dose of radiation was sufficient to decrease (*F_(3,12)_ = 12.07, p = 0.0006*) the proportion of CD11b^+^ myeloid cells at 7 (*p<0.05)* and 14 (*p<0.01*) days after radiation compared to sham, however this deficit returned to sham levels by day 28 (Fig. 1A). Interestingly, although there was a decrease in the overall population of myeloid cells, we observed a significant increase in the percentage of CD11b^+^ cells that were F4/80^+^, a marker of activated macrophages (*F_(3,12)_ = 4.048, p = 0.0335*), after radiation exposure. A pairwise comparison revealed significance only at the 7-day time point (*p<0.05)* compared to sham (Fig. 1B,D). Commensurately, we observed a trend for downregulation of CD11b^+^ cells that were F4/80^−^ (Fig. 1C,D).

### 10 Gy Cranial Irradiation is Sufficient to Induce the Migration of F4/80^+^CCR2^+^ Peripheral Macrophages into the Brain

**Figure 2:**
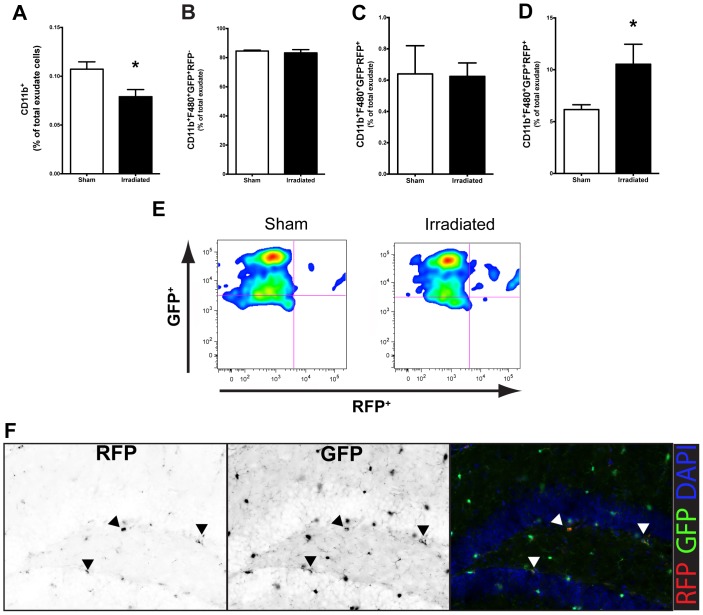
Cranial irradiation induces the infiltration of peripherally derived macrophages at seven days. **A.** The percentage of CD11b^+^GFP^+^ microglial cells was significantly decreased in *CCR2^RFP/+^CX3CR1^GFP/+^* irradiated mice compared to sham (Student’s t-Test; *p<0.05). **B.** Cranial irradiation does not affect the percentage of resident macrophages (CD11b^+^F4/80^+^CX3CR1^+^CCR2^−^, Student’s t-Test; *p>0.05*), **C.** nor the proportion of peripheral macrophages (CD11b^+^F4/80^+^CX3CR1^−^CCR2^+^, Student’s t-Test; *p>0.05*); **D.** but is associated with a significant increase in the percentage of infiltrated peripherally derived cells that had differentiated into microglia-like macrophages (CD11b^+^F4/80^+^CX3CR1^+^CCR2^+^, Student’s t-Test; *p<0.05). **E.** Representative plots showing the radiation-induced accumulation of peripherally derived macrophages into the brain parenchyma. **F.** Moreover, these cells can be seen in the dentate gyrus of the dorsal hippocampal formation following irradiation. RFP and GFP single pseudo-colored image is converted to black and white to discriminate location in the color co-localized panel. Black arrowheads point to individual positively labeled cells for their respective fluorchrome, while white arrowheads show the colocalized cells in the pseudo-colored image.

Recent evidence using a microglia “death signal” model, wherein all CNS microglia are systematically depleted, suggests that peripherally derived macrophages infiltrate into the CNS and replace resident microglia [40]. As defined by the expression of CCR2, our studies examined if peripherally derived macrophages infiltrate the CNS following cranial irradiation. Seven days after irradiation in *CCR2^RFP/+^CX3CR1^GFP/+^* reporter mice, our data showed that there is a depletion of CD11b^+^GFP^+^ cells (Fig. 2A, *p<0.05*); similar to what was observed in WT mice. There was no change in percentage of activated (F4/80^+^) CX3CR1^+^ cells in irradiated mice compared to sham (Fig. 2B). Additionally, while there were no differences in the proportion of infiltrated macrophages (F4/80^+^CCR2^+^) in response to cranial irradiation (Fig. 2C), there was a significant increase (*p<0.05*) in the percentage of cells that expressed both CCR2 and CX3CR1 (Fig. 2D,E) seven days after cranial irradiation. Notably, these double-labeled cells are visible in the dorsal HPC by immunofluorescence (Fig. 2F).

### Cranial Radiation Alters Microglia and Astrocyte Gene Expression in the Hippocampus

**Figure 3:**
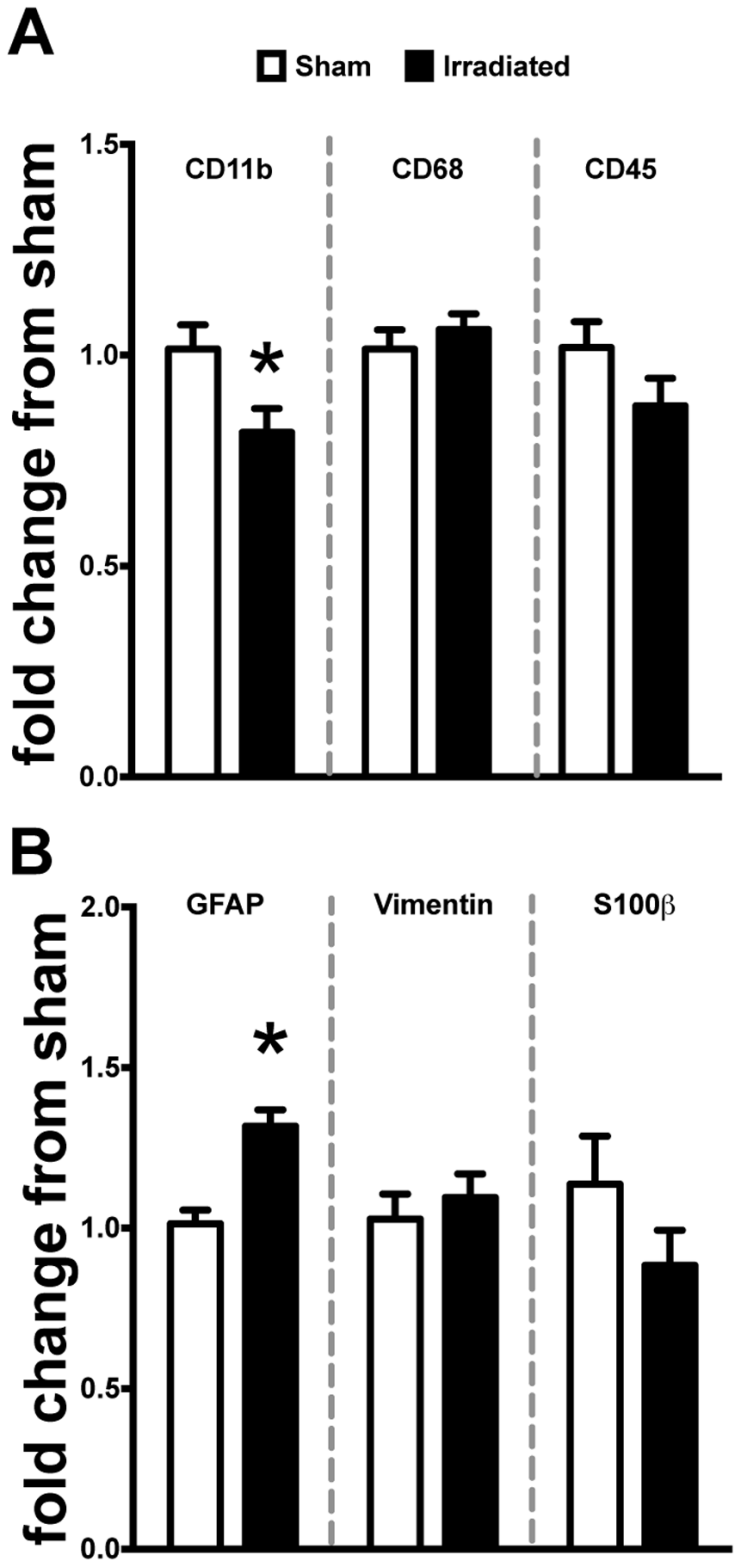
Cranial irradiation alters microglia and astrocyte molecular markers of activation in the hippocampus of WT mice. **A.** Irradiation induced a significant decrease of CD11b gene expression compared to sham (Student’s t-Test; *p<0.05) but no changes in the phagocytic or haematopoetic markers CD68 or CD45 (Student’s t-Test; *p>0.05*). **B.** GFAP expression was significantly increased compared to sham (Student’s t-Test; *p<0.05). Conversely, there were no changes in gene expression for alternate markers associated with astrocyte activation: vimentin and S100β (Student’s t-Test; *p>0.05*).

The hippocampus has been shown to be especially vulnerable to radiation-induced damage. Because of the observed changes in the myeloid cell population, we next examined HPC gene expression for markers of microglia and astrocyte reactivity. In accordance with our flow cytometry data in both WT and *CCR2^RFP/+^CX3CR1^GFP/+^* mice, we observed a significant decrease in *CD11b* mRNA expression compared to sham (Fig. 3A, *p<0.05*). However, there were no changes induced by radiation exposure to CD68, a lysosomal marker associated with phagocytic microglia/macrophages. We next examined CD45, which is a haemotopoetic marker of monocytes/macrophages but found no difference initiated by irradiation, although there was a trend for a reduction. Astrocytes have been shown to contribute to innate immune response by releasing multiple pro-inflammatory cytokines and chemokines [41]. Relative gene expression for glial fibrilarly acid protein (GFAP) was significantly induced in the HPC of irradiated mice compared to sham (Fig. 3B). However, when we examined vimentin and S100β as other markers associated with astrocyte activation we found no differences induced by irradiation.

### Cranial Irradiation Induces Pro-inflammatory Response in the Hippocampus of WT Mice

**Figure 4:**
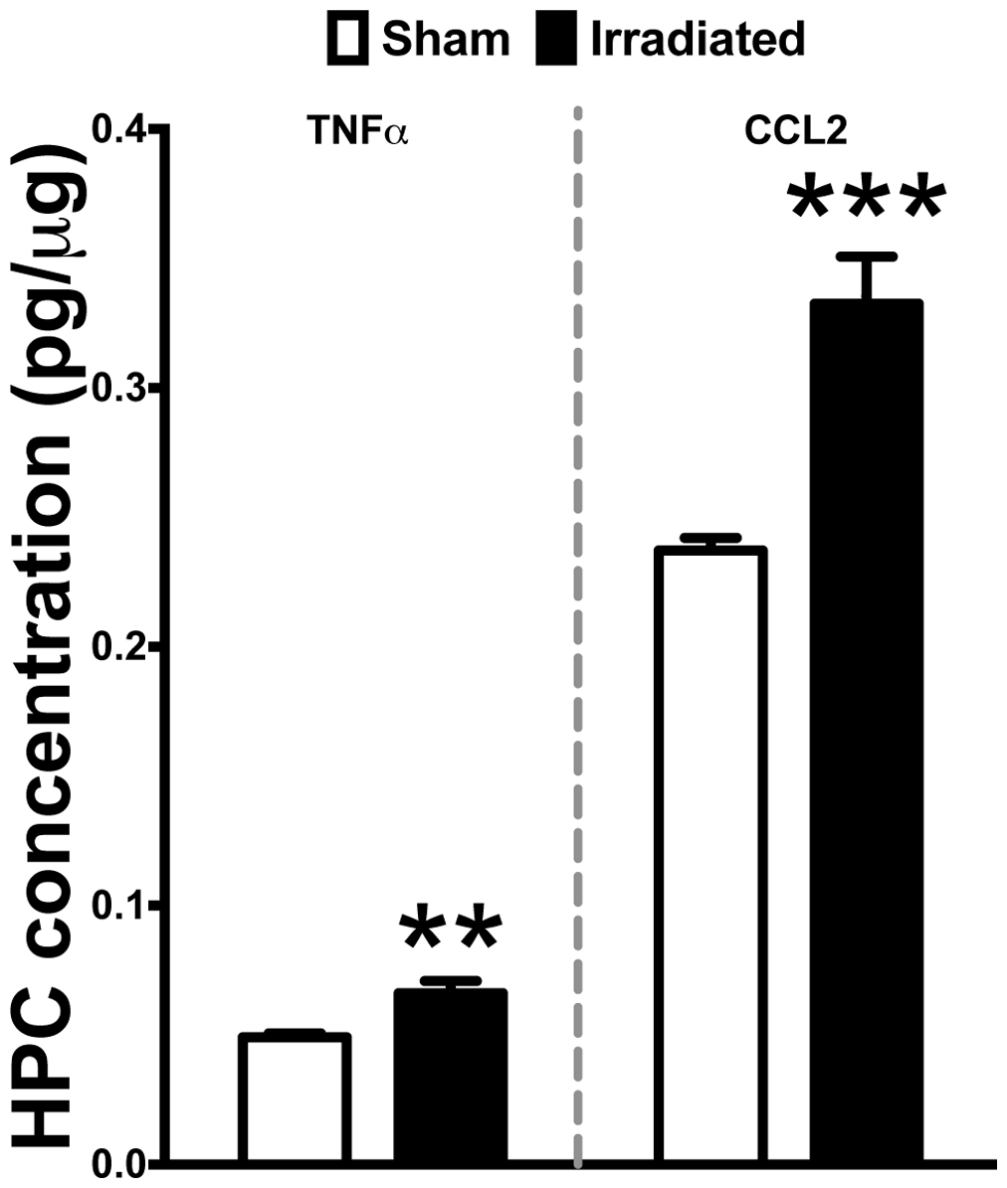
Cranial irradiation is associated with a significant increase of pro-inflammatory signaling molecules in the hippocampus. Standard ELISA analysis revealed a significant induction in TNFα (Student’s t-Test; **p<0.01) and CCL2 (Student’s t-Test; ***p<0.001) levels in the HPC of WT irradiated mice compared to sham.

Given our data showing multiple alterations in both the cell types and markers associated with innate immune function, we next examined two pro-inflammatory factors classically associated with these responses. Specifically, we measured the concentrations of TNFα and CCL2 from the hippocampus of WT mice seven days after cranial irradiation. Induction of TNFα has pleiotropic effects in the CNS, most notably protracted expression of this pro-inflammatory cytokine has been shown to be neurotoxic in a variety of animals models of disease. Moreover, increased expression of the pro-inflammatory chemokine CCL2 has been shown to chemotactically recruit CCR2^+^ macrophages to sites of inflammation [13], [42]–[45]. Herein, our data show that 10 Gy radiation exposure significantly upregulates the production of both TNFα (*p<0.01*) and CCL2 (*p<0.001;* Fig. 4). Interestingly, we did not observe any changes in mRNA gene expression of CCL7, CCL8, or CCL12 in the HPC of irradiated mice compared to sham (data not shown).

### 10 Gy Cranial Radiation does not Alter BBB Integrity but does Affect Multiple Signaling Cascades Associated with Endothelial Function in the HPC

**Figure 5:**
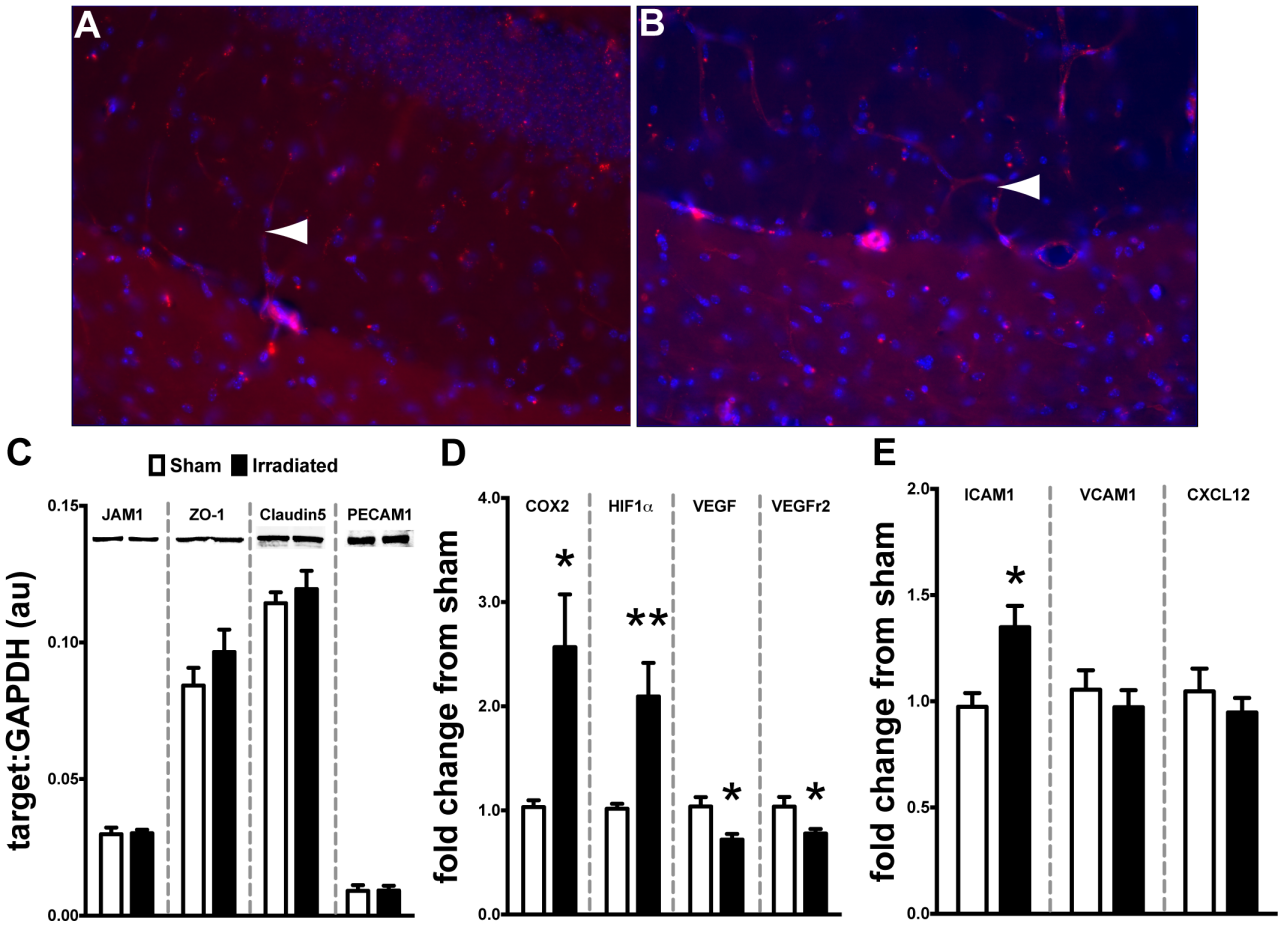
Cranial irradiation does not affect BBB integrity but does alter vascular signaling molecules. Tail-vein injected rhodamine-lectin remained within the vascular compartment without leaking into HPC parenchymal space in either the sham (**A**) or irradiated (**B**) treated mice seven days after irradiation. White arrowhead indicate positively stained rhodamine-lectin labeled microvasculature in the dorsal HPC. **C.** Complementing the lectin experiment, Western blot analysis revealed that 10 Gy of cranial irradiation was not sufficient to alter the expression of JAM1, ZO-1, Claudin5, or PECAM1, which are multiple proteins associated with BBB function (Student’s t-Test; *p>0.05*). **D.** There was a significant induction of signaling intermediates associated with vascular function: COX2 (Student’s t-Test; *p<0.05) and HIF1α (Student’s t-Test; **p<0.05) gene expression in the HPC of irradiated WT mice. Conversely, we observed a significant down regulation in gene expression of VEGF (Student’s t-Test; *p<0.05) and its cognate receptor VEGFr2 (Student’s t-Test; *p<0.05). **E.** Irradiation induced the expression of monocyte associated cellular adhesion molecule ICAM1 in the HPC (Student’s t-Test; *p<0.05) but not VCAM1 or CXCL12 (Student’s t-Test; *p>0.05*).

*In vitro* and *in vivo* data suggest that high doses of ionizing radiation can disrupt the physiological properties of the blood-brain barrier especially in light of increased CCL2 expression [46]–[51]. However, the effect of cranial radiation *in vivo* is not well defined. This dosage and time point failed to reveal any vascular leaking of tail-vein delivered lectin-B4 into the HPC parenchymal compartment (Fig. 5A/B). We also analyzed multiple molecular markers associated with the maintenance of BBB tight junctions, junction adhesion molecule 1 (JAM1), zona occludins 1 (ZO-1), claudin5, and platelet endothelial cellular adhesion molecule 1 (PECAM), using Western blot technique. At this dosage and time point there were no significant differences induced by radiation among any targets (Fig. 5C). Next, we examined multiple signaling molecules associated with inflammation and altered vascular response. Specifically, there was a significant induction of cyclooxygenase 2 (COX2) mRNA in the hippocampus of irradiated mice compared to sham (Fig. 5D, *p<0.05*); a similar increase was found in the expression of hypoxia inducible factor 1 alpha (HIF1α, *p<0.01*). Importantly, these signaling molecules have been implicated in the regulation of vasculogenesis [52]–[56] by altering the expression of vascular endothelial growth factor (VEGF) and its cognate receptor (VEGFr2). Our results demonstrate a significant decrease in both VEGF (*p<0.05*) and VEGFr2 (*p<0.05*) mRNA in the hippocampus of irradiated mice compared to sham. Ionizing radiation and inflammation have been shown to alter the expression of endothelial-associated adhesion molecules (e.g. integrins and chemokines), which may influence the arrest, adhesion, and migration of circulating immune cells (e.g. macrophages) into tissue compartments [57]–[59]. Lastly, we examined the expression of multiple molecules associated with the recruitment of peripheral macrophages into CNS parenchyma [58]. Irradiation resulted in a significant increase (*p<0.05*) in the expression of ICAM1 in the HPC compared to sham (Fig. 5E). However, no radiation-induced changes were observed for VCAM1 or CXCL12, which are both implicated in differential recruitment of peripheral monocytes [57], [58], [60].

## Discussion

Recently, we demonstrated that CCR2-deficient mice have reduced levels of irradiation-induced inflammatory response in the hippocampus, and improved cognitive function as compared to wild type animals. These results suggest that CCR2 plays an important role in radiation-induced hippocampal neuronal dysfunction [10], either directly or through the modulation of other pathways. Based on these results, we hypothesized that cranial irradiation may alter the brain’s microenvironment sufficiently to permit the infiltration of peripherally derived, pro-inflammatory CCR2^+^ macrophages. To test this hypothesis we used both WT and *CCR2^RFP/+^CX3CR1^GFP/+^* reporter mice [16] to examine the effects of cranial irradiation on the brain’s myeloid cell population; central and peripherally derived. Further, we examined the expression of multiple inflammation-related signaling molecules in the hippocampus to determine if these changes may mediate the infiltration of CCR2^+^ macrophages from systemic circulation following cranial radiation.

Overall, radiation exposure produced a persistent decrease in the percentage of CD11b^+^ microglia in WT mice, which returned to sham levels by 28 days after irradiation. Previous work in a rat model of lesioned spinal cord has shown that 7 days following 25 Gy exposure was sufficient to reduce the number of microglia in the lesioned, non-lesioned, and sham tissues exposed to radiation [61]. However, to the authors’ best knowledge, these are the first data showing that cranial irradiation is also capable of altering the brain’s resident microglia population within a relatively short time period. Although radiation induced an overall decrease in the proportion of resident microglia seven days following exposure, at this time point we observed a significant increase in the percentage expressing F4/80, a classic marker of macrophage activation [62]. Given these unexpected results at the seven-day time point, we extended these parameters to the *CCR2^RFP/+^CX3CR1^GFP/+^* mice to be able to fully delineate the effect of cranial irradiation upon both resident (GFP^+^) versus infiltrating (RFP^+^) microglia/macrophages. Corroborating the effects we observed in WT mice, we again found a significant decrease in the percentage of CD11b^+^GFP^+^ resident microglia in the irradiated mice at the seven-day time point.

By using *CCR2^RFP/+^CX3CR1^GFP/+^* mice, it is possible to distinguish resident microglia/macrophages from peripherally derived monocytes/macrophages [16]. Seven days following irradiation we found a significant increase in the proportion of macrophages (CD11b^+^F4/80^+^) that co-express GFP and RFP (CX3CR1^+^CCR2^+^). These findings suggest that cranial radiation is sufficient to induce the migration of peripherally derived macrophages into the brain parenchyma. Moreover, the expression of both CX3CR1 and CCR2 on the same cell suggest that these cells, originally of a peripheral origin (CX3CR1^−^CCR2^+^), start to share some feature of microglia, as resident microglia do not natively express CCR2 in naïve or diseased conditions. A caveat to our findings is that at this time point we were unable to find a significant change due to irradiation in the newly immigrated peripheral macrophages (CX3CR1^−^CCR2^+^). These findings may suggest that there may be an acute response to radiation exposure that induces peripheral macrophage immigration into the CNS prior to the seven-day time point we examined. Our findings are also corroborated by prior work in bone marrow chimeric mice, showing engraftment of donor-derived monocytes in the spinal cord following whole body irradiation [14]. Furthermore, it is possible that the radiation-induced migration and differentiation of peripheral macrophages represents an attempt to replace depleted resident microglia. Recent work has shown that selective depletion of resident microglia without blood brain barrier disruption creates a permissive environment for the recruitment, infiltration, and engraftment of peripheral macrophages [40].

Hippocampal function is especially sensitive to the effects of radiation exposure, ultimately resulting in a negative impact on learning and memory [2]–[4]. Given the cellular changes observed with flow cytometry analysis, we next examined the hippocampus of WT mice for multiple signaling molecules that have been consistently implicated in the migration of peripheral macrophages into damaged tissue. Confirming the changes we observed with decreased proportions of CD11b^+^ microglia/macrophages in flow cytometry, we found a significant decrease in gene expression levels of CD11b in the hippocampus of irradiated mice seven days after irradiation. Interestingly, there were no changes in other phenotypic markers of microglia/macrophage activation (CD68 and CD45). Recent work has shown substantial heterogeneity among macrophage populations and that F4/80 expression is present on the majority of tissue macrophages while other markers (e.g. CD68 and CD45) may represent only a minor fraction of the total population [63].

Astrocyte activation has been consistently shown to play a role in the innate immune response associated with various models of neurodegenerative disease [41], [59], [64]–[66]. Our study revealed a significant increase in GFAP gene expression in the hippocampus of irradiated mice, but not in vimentin or S100β as other markers of astrocytosis. Interestingly, we did observe a trend for decreased expression of S100β in irradiated mice. This decline may be due to the expression of S100β on oligodendrocytes and their precursors [67], which undergo apoptosis following irradiation [68].

Brain irradiation can induce the local expression of multiple cytokines in the rodent brain [7]. In accordance with previous findings, the present results show a significant induction of the pro-inflammatory cytokine TNFα and chemokine CCL2 in the hippocampus of irradiated mice. TNFα expression has pleiotrophic effects in the CNS, notably it has been shown to concomitantly induce the expression of CCL2 in astrocytes [34], [69]–[71]. Moreover, increased expression of CCL2 has consistently been shown to alter the integrity of the blood brain barrier [48], [50], [51], [72]. In the present study, we did not observe any radiation-induced effects on the expression of multiple proteins associated with endothelial tight junction integrity in the hippocampus. However, others have demonstrated vasculature vulnerability and rarefaction following irradiation [73]–[75] and these changes may be sufficient to induce multiple factors associated with the recruitment of systemic macrophages into damaged tissues [57], [59], [76], [77]. Specifically, we observed a significant increase in ICAM1 hippocampal gene expression. However, other markers associated with endothelial mediated macrophage recruitment were not altered at this time point. These findings are in agreement with the lack of change in the percentage of peripheral macrophages (CX3CR1^−^CCR2^+^) at this time point and may further suggest that the elevated levels of ICAM1 are a residual response.

Next, we examined upstream signaling mediators of the altered inflammatory and endothelial response. In the hippocampus of irradiated WT mice, there was a significant increase in the expression of two early response genes COX-2 and HIF1α. Prostaglandins, a COX-2 dependent by-product, have been shown to induce the expression of HIF1α in normoxic conditions, perpetuating inflammatory response [78]. Moreover, these two mediators have been shown to alter the expression of VEGF, a potent angiogenic factor for endothelial cells [53], [79]. Herein, we showed that both VEGF and its cognate receptor (VEGFr2) gene expression are significantly downregulated in the irradiated hippocampus. These findings are in agreement and extend previous work in a rat model of cranial irradiation that showed similar reductions in multiple early time points [54]. Taken together, the increased expression of pro-inflammatory early response genes COX-2 and HIF1α alongside the concomitant increase in TNFα and CCL2 may create a feed-forward pro-inflammatory loop ultimately resulting in hippocampal microvasculature rarefaction following radiation exposure. Consequently, this in turn could explain the increase cellular adhesion molecules driving the increased migration of peripherally derived macrophages to the brain parenchyma.

In conclusion, the present study demonstrates that of the brain’s innate immune response is particularly vulnerable to cranial irradiation. Alterations of the brain’s local microenvironment may be responsible for the recruitment and immigration of peripherally derived macrophages in an effort to replace apoptotic or dysfunctional resident microglia. Moreover, the infiltration of peripheral macrophages seems to be due to chemotactic signaling and not dependent on the disruption of the BBB. It remains unclear if peripherally derived macrophages can functionally substitute for resident microglia. Cumulatively, radiation-induced alterations in the production of cytokines, chemokines, and growth factors may contribute to altered endothelial function and upregulation of adhesion molecules implicated in the recruitment of peripheral macrophages from systemic circulation. All together, these data provides novel insight into a potential molecular mechanism that may contribute to radiation brain-injury. Further work is still needed to define the role of these infiltrating macrophages in response to radiotherapy.
